# Emergency Valve-in-Valve Transcatheter Aortic Valve Implantation for the Treatment of Acute Stentless Bioprosthetic Aortic Insufficiency and Cardiogenic Shock

**DOI:** 10.1155/2018/6872748

**Published:** 2018-03-13

**Authors:** Ivan D. Hanson, Pratik K. Dalal, Brian M. Renard, George S. Hanzel, Alessandro Vivacqua

**Affiliations:** ^1^Department of Cardiovascular Medicine, Beaumont Health System, Royal Oak, MI, USA; ^2^Department of Cardiovascular Surgery, Beaumont Health System, Royal Oak, MI, USA

## Abstract

Bioprosthetic aortic valve degeneration may present as acute, severe aortic regurgitation and cardiogenic shock. Such patients may be unsuitable for emergency valve replacement surgery due to excessive risk of operative mortality but could be treatable with transfemoral valve-in-valve transcatheter aortic valve implantation (TAVI). There is a paucity of data regarding the feasibility of valve-in-valve TAVI in patients presenting with cardiogenic shock due to acute aortic insufficiency from stentless bioprosthetic valve degeneration. We present one such case, highlighting the unique aspects of valve-in-valve TAVI for this challenging patient subset.

## 1. Introduction

Acute severe aortic regurgitation is an emergency necessitating immediate intervention due to the inability of the left ventricle to adapt to the sudden increase in preload and left ventricular diastolic pressure, which ultimately results in pulmonary edema and cardiogenic shock. Pharmacologic preload and postload reduction can temporize clinical deterioration, but only valve replacement can abort the inevitable hemodynamic collapse. We present a case of successful valve-in-valve transcatheter aortic valve implantation (TAVI) for a structurally deteriorated Medtronic Freestyle stentless bioprosthesis in a patient presenting with severe aortic regurgitation and cardiogenic shock. Due to clinical presentation in extremis and presence of stentless bioprosthetic degeneration, the case presented unique challenges that highlight important aspects of preprocedural care, heart team collaboration, imaging guidance, and implantation technique.

## 2. Case Report

**Figure 1:**
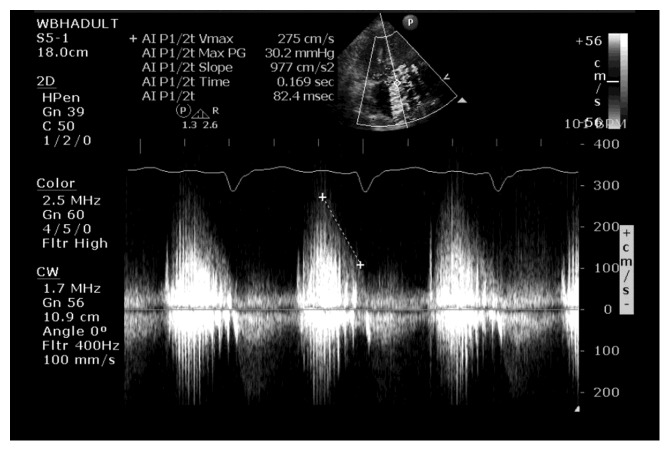
Transaortic Doppler demonstrating dense aortic regurgitation signal with markedly reduced pressure half time, suggestive of severe aortic insufficiency.

The patient is a 72-year-old female with a history of diabetes mellitus, hypertension, and bicuspid aortic valve status post aortic valve and root replacement using a 23 mm Freestyle (Medtronic, Minneapolis, MN) aortic valve and root bioprosthesis implanted by root inclusion method 12 years prior to admission. She was transferred from an outside hospital with acute-onset chest pain and shortness of breath. She denied fever and chills. Physical examination was notable for blood pressure 100/30 mmHg and hypoxia. Grade 3/6 systolic and diastolic murmurs were heard at the right 2nd intercostal space. Subsequently, she developed pulmonary edema and shock necessitating mechanical ventilation and vasopressor support. A transthoracic echocardiogram suggested flail aortic valve leaflet (Video 1), and transaortic Doppler signal was suggestive of wide-open aortic regurgitation (Figure 1).

Due to rapid deterioration of the patient, the heart team was immediately organized. Two cardiac surgeons deemed her unsuitable for redo surgical aortic valve replacement (Society of Thoracic Surgeons predicted operative mortality >50%). Therefore, valve-in-valve TAVI was considered. After review of the operative report and manufacturer product information, the inner diameter of the Freestyle valve was confirmed to be 20 mm. A 23 mm S3 valve (Edwards Lifesciences, Irvine, CA) was selected. Images from a remote contrast-enhanced CT scan suggested absence of peripheral arterial calcification and adequate caliber of iliofemoral arteries for large-bore sheath insertion.

**Figure 2:**
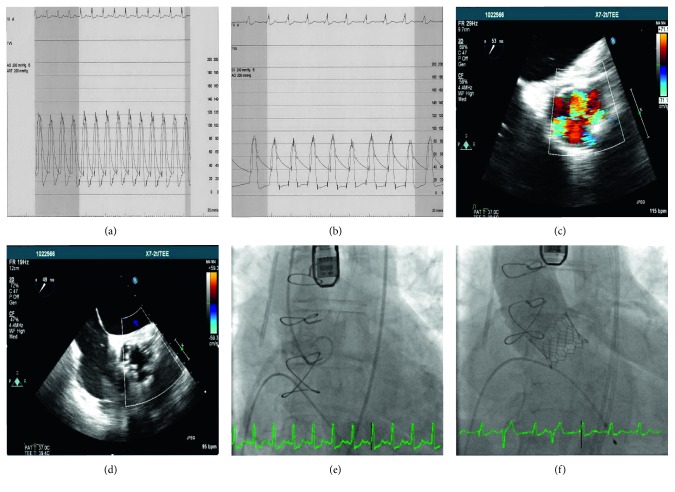
(a) Pre-TAVI simultaneous aortic and left ventricular pressures demonstrating equalization of Ao diastolic LV end-diastolic pressures. (b) Post-TAVI hemodynamics revealing normal diastolic LV-Ao gradient and no systolic LV-Ao gradient. (c) Pre-TEE image of the bioprosthetic aortic valve during diastole shows three large regurgitant jets. (d) Post-TEE short axis of the TAVI valve during diastole shows absence of regurgitant flow. (e) Pre-TAVI aortogram shows 4+ aortic insufficiency. (f) Post-TAVI aortogram shows no aortic insufficiency and acceptable implantation depth. TAVI = transcatheter aortic valve replacement; Ao = aortic; LV = left ventricular; TEE = transesophageal echocardiogram.

**Figure 3:**
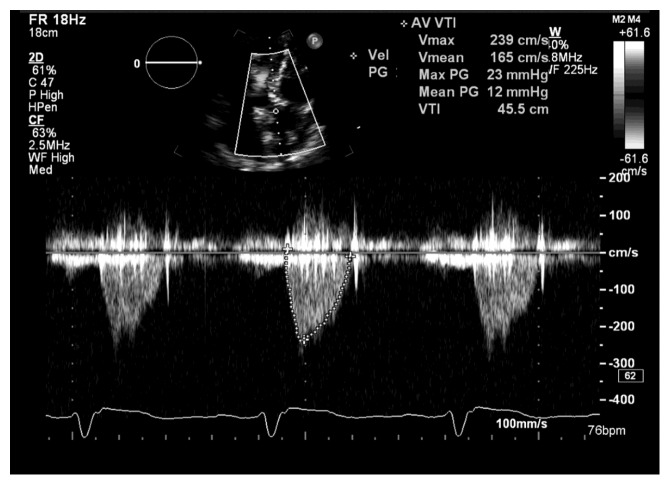
Transaortic Doppler at 30 days after TAVI reveals mean systolic gradient of 12 mmHg and no aortic insufficiency. TAVI = transcatheter aortic valve replacement.

Transesophageal echocardiography (TEE) was used to guide the procedure. The etiology of valve deterioration was confirmed to be leaflet flail; no vegetations were identified. Arterial and venous access was obtained followed by placement of a transvenous pacemaker. Aortography was performed (Video 2), followed by insertion of a 14 F × 36 cm E sheath (Edwards Lifesciences, Irvine, CA). The 23 mm S3 valve was implanted during rapid pacing, using nominal inflation volume (Video 3). Of note, wide pulse pressure was observed even during pacing at 200 beats per minute. Pre-TAVI central aortic pressure was 114/30 mmHg, and left ventricular pressure was 120/30 mmHg (Figure 2(a)). Post-TAVI central aortic pressure was 90/40 mmHg with a left ventricular pressure of 90/13 mmHg (Figure 2(b)). The final TEE images and aortogram showed trivial aortic regurgitation (Figures 2(c)–2(f)). Blood cultures, which had been obtained at presentation, were negative. The patient was discharged home in stable condition after 7 days. At a 30-day postprocedure follow-up visit, she was asymptomatic and had returned to working as a teacher. An echocardiogram at that time revealed normal left ventricular size and function, no aortic insufficiency, mean transaortic gradient 12 mmHg, and calculated aortic valve area 1.0 cm^2^ (Video 4, Figure 3).

## 3. Discussion

Several large, multicenter registries have demonstrated that valve-in-valve TAVI is safe and effective in stable patients with degenerated aortic bioprostheses [1–4]. In an analysis from the Global Valve-in-Valve Registry, Dvir and colleagues demonstrated excellent 1-year survival in such patients (83.2%; 95% CI, 80.8%–84.7%) [2]. One-year survival for patients with pure insufficiency as the mode of bioprosthetic failure was even more favorable (91.2%; 95% CI, 85.7%–96.7%). Of patients with pure insufficiency, 36.7% had New York Heart Association (NYHA) 4 symptoms (number of patients in shock was not reported), and 29.6% had stentless valve degeneration.

In contrast to more stable patients with failed *stented* bioprostheses, valve-in-valve TAVI for acute, severe aortic insufficiency and cardiogenic shock due to *stentless* bioprosthetic degeneration represents a particularly challenging TAVI subset, and published data are limited to a few case reports. Bagur and colleagues [5] treated a patient with 15-year old degenerated 23 mm Medtronic Freestyle (Minneapolis, Minnesota) who presented with hemodynamically unstable pulmonary edema. They used a 23 mm Edwards SAPIEN XT (Edwards Lifesciences Inc., Irvine, California). Since there was relative lack of calcification in the body of the prosthesis, valve positioning was facilitated by a pigtail catheter left in the right sinus of Valsalva until the SAPIEN XT was half deployed, and the operators used a very slow deployment technique. Chevalier and colleagues [6] treated a patient presenting with shock and acute aortic insufficiency due to a deteriorated 19 mm Freestyle valve with a 23 mm SAPIEN XT valve with 5 cc less than nominal inflation volume; a transcatheter valve with a smaller diameter was not used due to patient-prosthesis mismatch at baseline. The hemodynamic result was acceptable (shock resolved, residual 25 mmHg mean transaortic gradient with no aortic insufficiency), and the patient was free of heart failure at 6-month follow-up. Finally, Duncan et al. [7] reported a series of valve-in-valve TAVI in 22 patients with failing stentless bioprostheses, one of which had a failed 25 mm homograft and presented with acute aortic insufficiency and cardiogenic shock. Extracorporeal circulatory support was used before and after TAVI, which utilized a 29 mm Medtronic Corevalve (Minneapolis, Minnesota). The Corevalve was 4.4% oversized based on the perimeter of the homograft annulus. All patients in the series, including this one, were alive at 30 days.

Emergency valve-in-valve TAVI in a patient with *acute*, *severe aortic insufficiency* and *cardiogenic shock* presents unique technical challenges. Preprocedural stabilization in the crucial moments prior to TAVI must include airway intubation and hemodynamic support. Pharmacologic vasopressor support may be necessary in the short term to increase central aortic pressure and perfuse vital organs. Intra-aortic balloon pump and transaortic axial flow pumps (such as Impella (Danvers, Massachusetts)) are generally contraindicated. Extracorporeal membrane oxygenation may be effective as a temporizing measure, as was reported by Duncan et al. [7], but an arterio-venous circuit in a beating heart would be expected to ultimately lead to worsening aortic regurgitation and pump failure [8]. Therefore, timely aortic valve replacement is the key to survival. With the airway secured, TEE can confirm the etiology of leaflet degeneration (i.e., whether due to a purely mechanical problem or endocarditis), visualize the sewing ring of the stentless valve (invisible by fluoroscopy), and may be helpful in coordinating the appropriate TAVI implant height. Preprocedural CT angiography, while ideal for planning stentless (or stented) valve-in-valve TAVI in more stable patients, is not feasible for patients in shock. Transfemoral access is the least invasive and likely to be the best tolerated large-bore vascular access for hemodynamically unstable patients. Although low mean systemic blood pressure was achieved with pacing at 200 bpm, pulse pressure remained relatively wide. This was likely the result of hyperdynamic ventricular contractility and stroke volume due to inotropic agents and severe aortic insufficiency, respectively. Despite this, the balloon-expandable transcatheter valve remained in a stable position throughout deployment.

In contrast to *stented* aortic prostheses, *stentless* prostheses do not have radiopaque markings to fluroscopically guide transcatheter valve implantation depth. The exception may be if there is heavy calcification in the body of the prosthesis. Importantly, the sewing ring may be more ventricular than the calcium landmarks in the body of the prosthesis, so a combination of TEE and aortography, with one or two pigtail catheters to locate the sewing ring relative to the root, may be useful [9]. For stable patients with stentless valve degeneration, self-expanding, recapturable transcatheter valves have been successfully used for valve-in-valve TAVI [10–12]. However, balloon-expandable valves have also been used with good results [5]. In the present case, oversizing of the S3 valve relative to the Freestyle inner diameter (23 mm versus 20 mm, resp.) provided some reassurance that device migration or embolization would be unlikely. Furthermore, baseline echocardiography and aortography (in lieu of CT angiography) suggested that the sinuses of Valsalva appeared to be sufficiently spacious and the height of the coronary ostia from the annulus appeared grossly adequate to accommodate the 23 mm S3 valve without causing coronary artery obstruction from the degenerated prosthetic valve leaflets. Fortunately, the aortogram (double the normal contrast volume was used for the injection) nicely opacified the sewing ring of the Freestlye prosthesis which was approximately 2 mm below the bottom of the pigtail catheter in the aortic root. Alignment of the middle marker of the S3 balloon catheter with this plane resulted in perfect implantation depth.

## 4. Conclusion

We present a challenging case of emergency valve-in-valve TAVI in a stentless bioprosthesis for a patient presenting with severe aortic regurgitation and cardiogenic shock using a balloon-expandable transcatheter valve. This case adds to the experience of the few reported cases of valve-in-valve TAVI for this uniquely challenging patient subset. Prompt diagnosis, preprocedural airway and hemodynamic stabilization, immediate heart team collaboration, and skillful use of invasive and noninvasive imaging were all instrumental in achieving a successful outcome.

## Supplementary Materials

Supplementary 1 Video 1: surface echocardiogram revealing failed bioprosthetic aortic valve leaflet.

Supplementary 2 Video 2: initial aortogram showing severe (4+) aortic insufficiency. Note the location of the bioprosthesis sewing ring approximately 2 mm below the bottom of the pigtail catheter.

Supplementary 3 Video 3: implantation of 23 mm S3 valve during rapid ventricular pacing.

Supplementary 4 Video 4: echocardiogram at 30 days after TAVI reveals stable valve-in-valve aortic prosthesis with no aortic insufficiency. TAVI = transcatheter aortic valve replacement.
